# Educational performance and conduct problem trajectories from childhood to adolescence: Observational and genetic associations in a Brazilian birth cohort

**DOI:** 10.1002/jcv2.12105

**Published:** 2022-10-17

**Authors:** Thais Martins‐Silva, Andreas Bauer, Alicia Matijasevich, Iná Santos, Aluísio Barros, Ulf Ekelund, Luciana Tovo‐Rodrigues, Joseph Murray

**Affiliations:** ^1^ Human Development and Violence Research Centre (DOVE) Federal University of Pelotas Pelotas Brazil; ^2^ Post‐Graduate Program in Epidemiology Federal University of Pelotas Pelotas Brazil; ^3^ Departamento de Medicina Preventiva Faculdade de Medicina FMUSP Universidade de São Paulo São Paulo Brazil; ^4^ Postgraduate Program in Pediatrics and Child Health School of Medicine Pontifical Catholic University of Rio Grande do Sul Porto Alegre Brazil; ^5^ International Center for Equity in Health Federal University of Pelotas Pelotas Brazil; ^6^ Department of Sport Medicine Norwegian School of Sport Sciences Oslo Norway; ^7^ Department of Chronic Diseases and Ageing Norwegian Institute of Public Health Oslo Norway

**Keywords:** conduct disorders, education, gene‐environment interaction, genetics, longitudinal studies

## Abstract

**Background:**

Educational difficulties are an important potential influence on both the onset and course of children's conduct problems. This study evaluated the association between school failure and children's conduct problems in Brazil, a context with high rates of both conditions, using both observational and genetic approaches.

**Methods:**

Prospective, population‐based, birth cohort study in Pelotas city, Brazil. Parents reported on conduct problems four times between ages 4–15 years, and group‐based trajectory analysis was used to classify 3469 children into trajectories of childhood‐limited, early‐onset persistent, adolescence‐onset, or low conduct problems. School failure was measured as having repeated a school grade up to age 11, and a polygenic risk score (PRS) predicting educational attainment was calculated. Multinomial adjusted regression models were used to estimate the association between school failure (observational measure and the PRS) and conduct problem trajectories. To consider possible variation in effects of school failure by social context, interactions were tested with family income and school environment (using both observational and PRS methods).

**Results:**

Children repeating a school grade had increased odds of being on to childhood‐limited (OR: 1.57; 95% CI 1.21; 2.03), adolescence‐onset (OR: 1.96; 95% CI 1.39; 2.75), or early‐onset persistent trajectory (OR: 2.99; 95% CI 1.85; 4.83), compared to the low conduct problem trajectory. School failure also predicted increased risk for early‐onset persistent problems versus the childhood‐limited problems (OR: 1.91; 95% CI 1.17; 3.09). Using a genetic PRS approach, similar findings were observed. Associations varied according to the school environment: school failure had larger effects on children in better school environments.

**Conclusion:**

School performance, whether measured in terms of repeating school grades or genetic susceptibility, was consistently associated with trajectories of child conduct problems into mid‐adolescence. We also found a larger association for children in better school environments.

## Graphical abstract

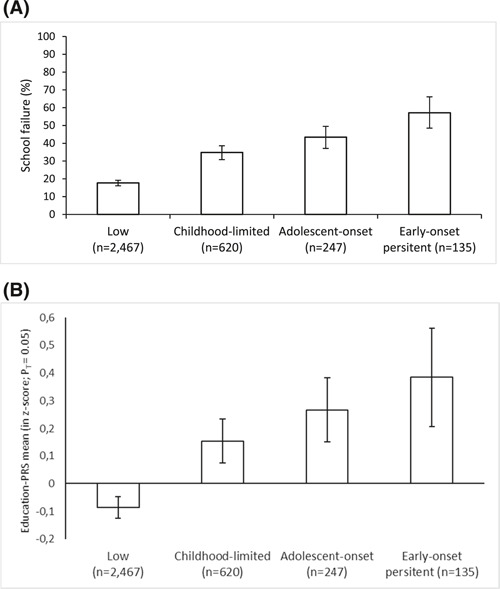

**Key points**

- In a large, longitudinal study in Brazil, school failure was strongly associated with conduct problems persisting through childhood into adolescence.
- Associations with school failure were smaller for conduct problems characterised as ‘childhood‐limited’ and ‘adolescent‐onset’.
- Findings were robust across two different methodological approaches: using statistical adjustment for covariates, and polygenic risk scores predicting educational attainment.
- Repeating a grade was more strongly associated with conduct problem trajectories among children in more favourable school environments.

## INTRODUCTION

Several, now classic, longitudinal studies in the second half of the 20th Century revealed a strong association between academic underachievement and elevated child conduct problems (see Maguin & Loeber, 1996; Murray & Farrington, 2010, for reviews). Nonetheless, methodological difficulties posed a significant challenge to identify the true impact of education on behaviour (Hinshaw, 1992), and current evidence is still inconclusive on whether a causal relation exists between poor educational performance and conduct problems, in either direction (Kulkarni et al., 2021). Moreover, despite the importance of person‐environment interactions in developmental psychopathology (Rutter et al., 1997), it is unclear whether any effects of educational performance on conduct problems vary by social context.

Given the complex bio‐psycho‐social determination of behaviour problems, isolating the contribution of poor educational performance is a major challenge (Fairchild et al., 2019). Education and behaviour are each influenced by both multiple environmental factors and genetics that could confound any association between them (Allegrini et al., 2020). Traditionally, studies have used statistical adjustment to adjust for measured environmental confounds. Wertz et al. (2018) recently suggested the use of polygenic risk scores (PRS) as a novel approach to testing the relationship between educational performance and conduct problems, providing triangulation with results from simple statistical adjustment. Over the last decade, GWAS has permitted the analysis of thousands of genetic variants to produce what are known as PRS, predicting a particular phenotype. A PRS summarises cumulative information across the entire genome to predict a particular condition. The first major success of this endeavour in the social sciences was the creation of a PRS predicting educational attainment among 100 thousand individuals (Rietveld et al., 2013), and this has been updated in multiple cohorts (Lee et al., 2018; Okbay et al., 2022).

A PRS genetic propensity affecting education should be less associated with other confounding factors (both environmental and genetic), than a measure of actual school performance. Hence, testing whether an education PRS predicts conduct problems can help strengthen causal inference. In the only study to date using this approach, Wertz et al. (2018) constructed a PRS for educational performance in the Dunedin longitudinal study in New Zealand, and the E‐Risk cohort in the UK, and related that to both poor educational attainment in mid‐adolescence and measures of conduct problems through the life‐course. They found that an education PRS predicted both crime and early‐onset persistent conduct problems across both samples. Further tests in other samples are needed, particularly using the PRS approach to compare with findings from traditional statistical adjustment methods in the same sample.

Poor educational performance may contribute to the development of conduct problems via multiple mechanisms. According to strain theories in criminology, frustration with school failure may lead to negative emotions affecting aggressive behaviour (Agnew, 1992) and may also influence cognitive and executive function skills involved in conduct problems (Moffitt, 1993a). Moreover, social bonding theory points to the importance of attachments to prosocial institutions, including school, protecting against conduct problems (Hirschi, 1969). Given multiple possible mechanisms involving social change after poor school performance, it seems especially likely that school failure could influence conduct problems when grade retention occurs—when a rupture of social ties is implicated, in addition to more direct, psychological consequences for children having academic difficulties.

Conduct problems themselves might also contribute to poor educational performance, but current evidence is unclear on whether bidirectional effects exist (Kulkarni et al., 2021). Although educational and behavioural difficulties may reinforce each other, the nature of this relationship could also vary according to different developmental trajectories of conduct problems. According to Moffitt's influential theory (Moffitt, 1993b, 2018), conduct problems are best understood when specified by their longitudinal course — according to whether they start early in childhood, whether they persist or decline thereafter, or first become elevated from adolescence onwards. Each trajectory may involve different mechanisms, distinguished either qualitatively or quantitatively (Fairchild et al., 2013). According to this model, it seems most likely that poor school performance and conduct problems become intertwined for children with elevated neuropsychological and behavioural difficulties early in life. Little evidence is available on this question, but two cohorts from New Zealand and the UK both supported the hypothesis that poor educational performance is most strongly linked to early‐onset persistent conduct problems, compared to other trajectories (Odgers et al., 2008; Wertz et al., 2018). In addition to common genetic risk factors, Carlisi et al. (2020) provided initial evidence that there are differences in brain surface morphometry in individuals with life‐course‐persistent conduct problems, a feature which may also partly explain their educational difficulties.

Despite improvements in recent years, Brazil is a country with high levels of school failure (UNICEF Brasil, 2021) and major challenges regarding youth conduct problems leading to serious crime (Murray et al., 2015). However, is no information regarding the conduct problems trajectories for Brazilian samples and how the educational performance impact each group. In the current study, we aim to estimate the effects of educational performance on children's conduct problem trajectories in a population‐based birth cohort in Brazil, using both observational and genetic approaches. Neither method is likely to be completely unbiased on its own but using both approaches in the same study provides a valuable point of triangulation to test the robustness of associations. We test the hypothesis that school failure (measured both observationally, and via a PRS for education) predicts increased conduct problems, especially manifest as early‐onset and persistent from childhood to adolescence. Finally, we also investigate possible variations in these effects according to the child's family income and school environments.

## METHODS

### Participants

The 2004 Pelotas Birth Cohort is a population‐based birth cohort of all children born between January 1 to December 31, 2004, in the city of Pelotas (340,000 inhabitants) in southern Brazil. Of 4263 mothers invited to participate when their children were born, 4231 (99.3%) gave informed consent and were included in the study with their children. Participants were re‐assessed at ages 24 months (*n* = 3869, 91.4% retention), 48 months (*N* = 3799, 89.8%), 6 years (*n* = 3669, 86.7%), and 11 years (*n* = 3565, 84.3%). At 15 years, we interviewed 50.4% of the cohort (*n* = 2029), before fieldwork was prematurely ended in March 2020 due to the Covid‐19 pandemic. 3469 participants (82.0% of the original cohort) were included in the final analyses of both approaches, after excluding participants with missing conduct problem trajectories (*n* = 293) and genetic information (*n* = 759). The cohort methodology has been described in detail elsewhere (Santos et al., 2014) and details are also included in Supporting Information S1.

### Measures of conduct problem trajectories

Conduct problem trajectories from ages 4–15 years were previously estimated, using repeated measures of conduct problems reported by the mother and transformed to *z*‐scores. Trajectories could be constructed for the vast majority of the cohort (*n* = 3938; 51.9% male and 48.1% female), with valid conduct problem information at 4, 6, 11 and 15 years (*n*s of 3750, 3580, 3563, and 1942, respectively). Note that, only around half of the participants were assessed at the age of 15 years. At age 4 years, conduct problems were measured using the parent‐rated Child Behaviour Checklist (CBCL) (Achenbach, 1991), which has been validated for use with Brazilian children (Bordin et al., 1995). The CBCL consists of 118 behavioural and emotional items, which are divided into eight subscales. The aggressive behaviour and rule‐breaking behaviour subscales were summed to derive a composite measure of conduct problems (ranging from 0 to 52). At ages 6–15 years, we used the parent‐rated Strengths and Difficulties Questionnaire (SDQ; ranging from 0 to 10) (Goodman, 2001). The SDQ has been validated for use in Brazil and correlates strongly with CBCL conduct problems (Saur & Loureiro, 2012). Group‐based trajectory modelling, a semi‐parametric approach (Nagin, 2005), was used to previously estimate conduct problems trajectories from 4 to 15 years (Martins‐Silva et al., under review).

### Measures of school failure and education‐PRS

School failure was measured at age 11 years, asking carers whether children had ever failed a grade (*yes* or *no*). A PRS for poor educational performance (hereafter called education‐PRS) was based on DNA information collected and analysed from saliva (see details in Supporting Information S1). The education‐PRS was based on a recent GWAS meta‐analysis of educational attainment based on 1,131,881 individuals of European ancestry, for whom 1271 independent SNPs were identified (Lee et al., 2018). Although we examined failure at school up to age 11 years, rather than later educational attainment, two cohort studies have found that a similar education‐PRS predicts both (Wertz et al., 2018).

To identify the most appropriate education‐PRS, we used the approach based on the clumping and thresholding methods due to its wide usage (Ni et al., 2021). We calculated four possible PRS‐education for each individual in the target sample as the sum of the SNPs weighted by their associations with education in the discovery study (Lee et al., 2018). Different *P*‐value cut‐off thresholds (*P*_*T*_) were used to define whether an SNP was included in the four PRS scores: *P*_*T*_ = 5 × 10^−8^; *P*_*T*_ = 5 × 10^−6^; *P*_*T*_ = 0.05 and *P*_*T*_ = 0.5 (Euesden et al., 2014). We adjusted for multiple comparisons by using the false discovery rate (FDR) to control possible Type I error. All four PRS‐education scores were reverse‐coded and transformed into *z*‐scores—so that higher scores indicate a higher genetic risk for poor educational performance. The resulting education‐PRS with the strongest association with school failure in this cohort was used in the main analysis (see Table S2).

### Covariates

When analysing carer‐reports of school failure as a predictor of conduct problem trajectories, we adjusted for child sex (observed at birth; male or female), and the following covariates measured at age 48 months: maternal schooling (0–4, 5–8, or ≥9 years), family income (tertiles), maternal depression, poor child development, and child attention problems. Maternal depression was measured using the Edinburgh Postnatal Depression Scale (EPDS) (Cox et al., 1987). The EPDS is a self‐report questionnaire, asking about depressive symptoms over the past 7 days and has been cross‐culturally adapted and validated for use in Brazil (Santos et al., 2007). The 10 items are scored on a 3‐point scale (ranging from 0 to 30). Battelle's Development Inventory (BDI) (Newborg, Stock, & Wnek, 1988) is a screening tool for child development comprising 96 items. We dichotomised the BDI scores to define a low child development group using the 10th percentile as the cut‐off point (i.e. children with low child development belonging to the first decile), given the relevance of this group in prior research in this sample (Barros et al., 2010). We also used the attention problems subscale of the CBCL (ranging from 0 to 16) (Achenbach, 1991). At age 6 years, the intelligence quotient (IQ) was measured, using the Wechsler Intelligence Scale for Children‐III (Barros et al., 2010). Low IQ was defined as a score of ≤70.

At age 11, we used information about the school environment as both a covariate and effect modifier. The school environment was assessed using 11 questions concerning the child's school and their relationships with classmates and teachers (including both positive and negative aspects; e.g., ‘Kids in my classroom push and shove each other a lot’ and ‘I feel safe at my school’). Items were chosen and adapted from the Peace Zone questionnaire (Prothrow‐Stith et al., 2001; For more details see Supporting Information S1). Items on school violence were reverse‐coded, so the final score (ranging from 0 to 33) indicated a more supportive, less violent school environment. As a covariate, the continuous score was used, and in moderator analysis, a categorical variable (in tertiles) was used.

When analysing education‐PRS, we include child sex and the first 10 principal components analysis (PCA) of ancestry. The PCA was based on the whole genomic dataset and was performed using PLINK1.9 (Purcell et al., 2007).

### Statistical analysis

First, missing data analyses were conducted comparing children included in the current analyses versus children lost to follow‐up on covariates, using Pearson's chi‐square tests and *t*‐tests. Second, we examined the association between carer‐reported school failure (repeating a school grade) and conduct problem trajectories, using adjusted multinomial logistic regression. Results are presented as odds ratios with 95% confidence intervals. All variables were entered simultaneously in the adjusted analyses. In sensitivity analyses, we re‐ran the models to see if associations remained when also adjusting for pre‐school behaviour problems, measured on the CBCL at age 4 years.

Next, we examined associations between education‐PRSs (based on different *P*_*T*_ values) and carer‐reported school failure, using logistic regression analysis. To identify the variance explained by each education‐PRS, a pseudo‐R‐squared (*R* ^2^) was estimated by calculating the difference between the full model (including the relevant education‐PRS and covariates) and the null model (covariates only). Using the best education‐PRS identified in our sample, we examined its associations with conduct problem trajectories, using adjusted multinomial logistic regression. In the adjusted model we included family income, maternal schooling, the school environment score, sex of the child, and 10 first principal components for genetic ancestry.

Finally, we tested whether associations between educational performance and conduct problem trajectories varied according to the following possible modifiers: family income and school environment, using the likelihood ratio test—comparing logistic regression models with and without relevant interaction terms. Analyses were performed with PRSice 2.2.1, STATA 16.1, and the RStudio software, and the code used is available at: https://github.com/ThaisMartins‐Silva/Educational‐performance‐and‐conduct‐problem‐trajectories‐code.git.

## RESULTS

**FIGURE 1:**
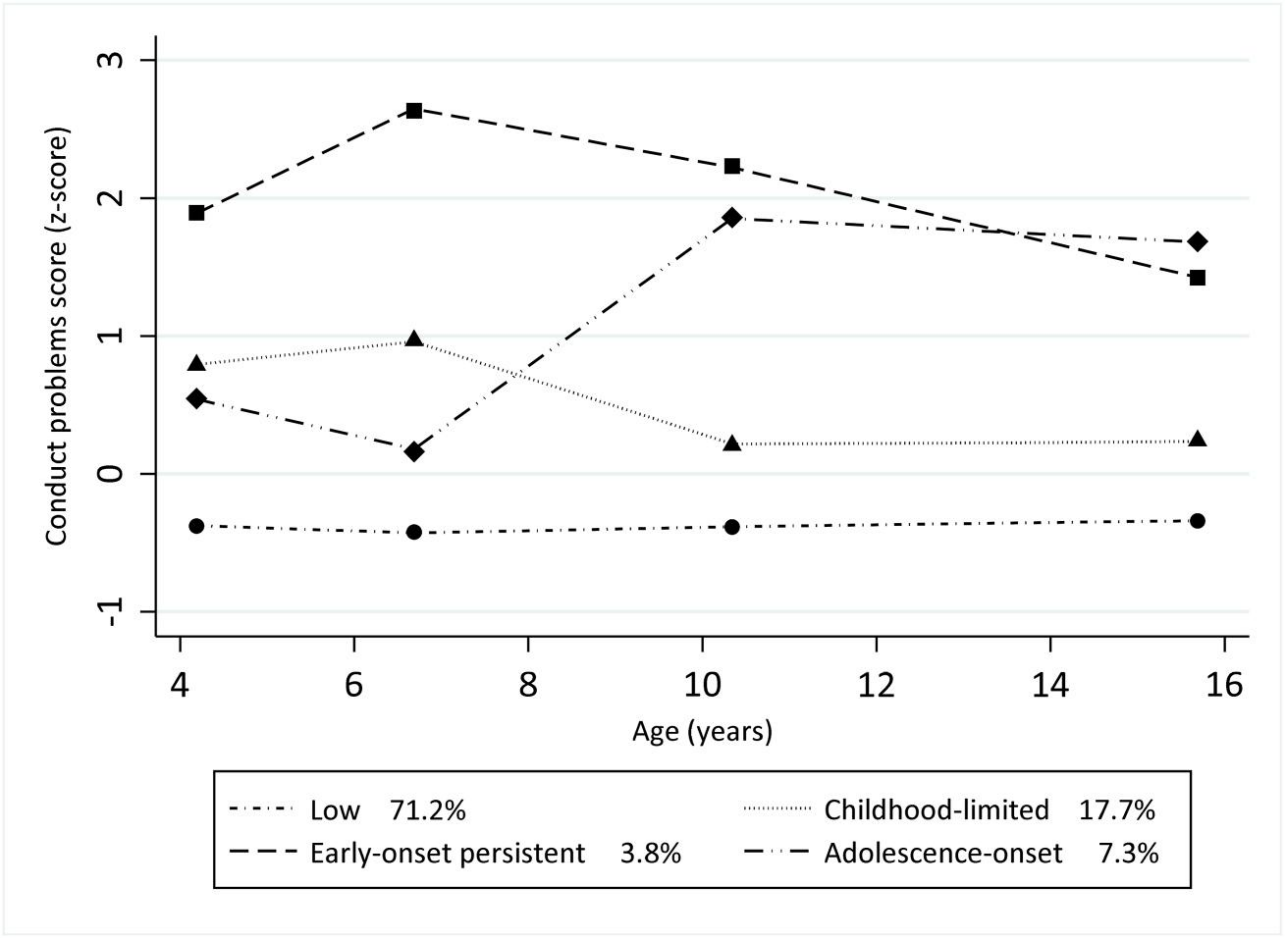
Conduct problem trajectories from ages 4 to 15 years in the 2004 Pelotas (Brazil) Birth Cohort. Lines represent estimated (latent) change over time. Dots represent observed group means

Conduct problem trajectories identified were shown in Figure 1 and descriptive statistics are presented in Table 1. We identified four groups of conduct problems: *early‐onset persistent*, in which conduct problems emerge in childhood and persist into adolescence; *childhood‐limited*, in which conduct problems remit in the transition from childhood to adolescence; *adolescence‐onset*, in which conduct problems first emerge in adolescence; and *low* conduct problems (see Figure 1 and Table S1). Almost one‐fourth of children (24.2%) failed a school grade by age of 11 years and nearly one‐fifth (17.9%) were classified as having childhood‐limited conduct problems, whereas a smaller proportion had adolescence‐onset (7.1%) and early‐onset persistent conduct problems (3.9%). Children in the analytic sample were less likely to have low IQ and had slightly lower levels of attention problems compared to those excluded (see Table 1).

**TABLE 1:** Description of sample and comparison between participants included and not included in the analysis

	Included (*n* = 3469)		Not included (*n* = 759)		*p*‐value^a^
	*N*	% (95% CI)	*n*	% (95% CI)	
Child sex					.089
Male	1780	51.3 (49.6; 52.9)	415	54.7 (51.1; 58.2)	
Female	1692	48.7 (47.1; 50.4)	344	45.3 (41.8; 48.9)	
Maternal schooling (in years)					.062
0–4	499	14.9 (13.7; 16.1)	82	19.1 (15.6; 23.1)	
5–8	1258	37.5 (35.9; 39.2)	147	34.2 (29.9; 38.8)	
≥9	1596	47.6 (45.9; 49.3)	201	46.7 (42.1; 51.5)	
Family income (in tertiles)					.561
1st (poorest)	1184	35.2 (33.6; 36.8)	159	36.9 (32.4; 41.6)	
2nd	1069	31.7 (30.2; 33.3)	126	29.2 (25.1; 33.7)	
3rd (richest)	1115	33.1 (31.5; 34.7)	146	33.9 (29.6; 38.5)	
Maternal depression					.259
Mean (SD)	3353	7.4 (5.48)	395	7.7 (5.75)	
School environment score^b^					.853
1st (better)	1190	37.0 (35.3; 38.)	114	37.9 (32.6; 43.5)	
2nd	1039	32.3 (30.7; 33.9)	99	32.9 (27.8; 38.4)	
3rd (worse)	992	30.8 (29.2; 32.4)	88	29.2 (24.4; 34.6)	
Low child development					.075
No	3015	89.6 (88.5; 90.6)	397	92.3 (89.3; 94.5)	
Yes	351	10.4 (9.4; 11.5)	33	7.7 (5.5; 10.6)	
Low IQ					.013
No	2350	70.4 (68.9; 72.0)	123	62.1 (55.2; 68.6)	
Yes	986	29.6 (28.0; 31.1)	75	37.9 (31.4; 44.8)	
Attention problems subscale					<.001
Mean (SD)	3346	2.6 (2.28)	403	3.0 (2.68)	
School failure					.067
No	2450	75.9 (74.3; 77.3)	214	71.1 (65.7; 75.9)	
Yes	780	24.2 (22.7; 25.7)	87	28.9 (24.1; 34.3)	
Conduct problems trajectories					.598
Low	2467	71.1 (69.6; 72.6)	338	72.1 (67.8; 75.9)	
Adolescent‐onset	247	7.1 (6.3; 8.0)	39	8.3 (6.1; 11.2)	
Childhood‐limited	620	17.9 (16.6; 19.2)	77	16.4 (13.3; 20.1)	
Early‐onset persistent	135	3.9 (3.3; 4.6)	15	3.2 (1.9; 5.2)	

*Note*: 2004 Pelotas (Brazil) Birth Cohort.

Abbreviations: 95% CI, 95% confidence interval; IQ, intelligence quotient; SD, standard deviation.

^a^ Pearson's chi‐square and *t*‐test analysis.

^b^ In tertiles.

### Education‐PRS and school failure

We examined four different education‐PRS (based on different *P*_*T*_ thresholds) in relation to school failure. All were associated with school failure, even after FDR correction. As expected from previous GWAS studies, the higher (inverted) education‐PRS, the greater the risk of school failure. The education‐PRS that had the strongest association with reported school failure (*P*_*T*_ = 0.05, 67,668 SNPs) explained 1% of the variance (pseudo‐*R* ^2^ = .0102, OR: 1.43; 95% CI: 1.27; 1.60) and was used in the main analyses (see Table S2).

### School failure, education‐PRS, and conduct problem trajectories

**FIGURE 2:**
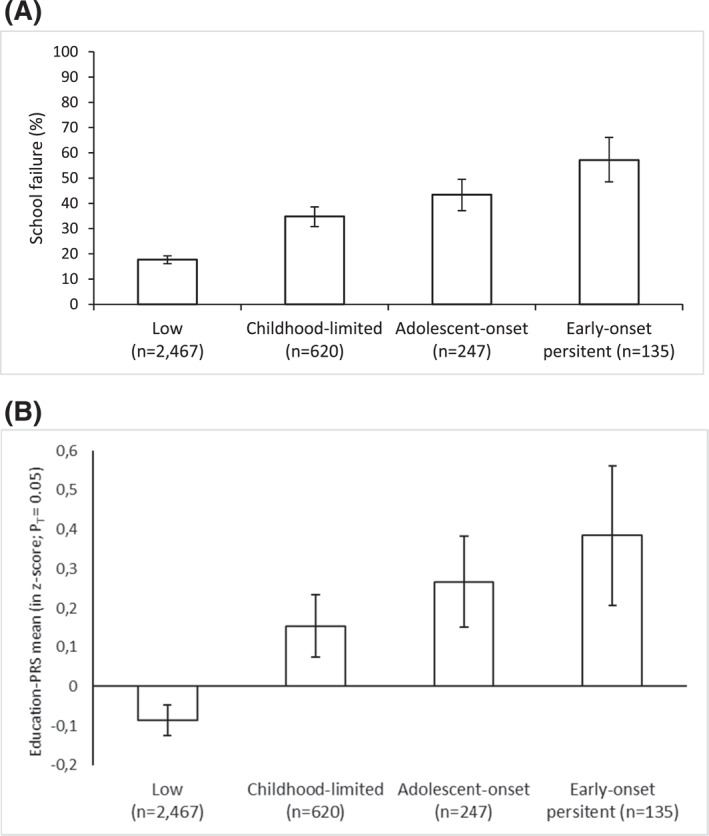
The proportion of children failing school by conduct problem trajectory group (A) and mean education‐PRS *z*‐score by conduct problem trajectory group (B)

Figure 2 shows line graphs of the relationship between conduct problem trajectories and: (Figure 2A) school failure, and (Figure 2B) mean levels of education‐PRS. A risk hierarchy can be observed such that children on the early‐onset persistent trajectory present the highest school failure and education‐PRS, followed by children in the adolescence‐onset, childhood‐limited, and the low groups.

Table 2 shows adjusted associations between school failure and conduct problem trajectories (Model A), and sensitivity analyses (Model B). Children who repeated a grade had increased odds of being on the childhood‐limited (OR: 1.57; 95% CI: 1.21; 2.03), adolescent‐onset (OR: 1.96; 95% CI: 1.39; 2.75), and early‐onset persistent (OR: 2.99; 95% CI: 1.85; 4.83), compared to the low trajectories (all *p* < .05; Model A). Repeating a grade also distinguished children in the early‐onset persistent group from those in the childhood‐limited group (OR: 1.91; 95% CI: 1.17; 3.09), but results were not significant when comparing the early onset‐persistent to the adolescence‐onset group. When behaviour problems at age 4 years were added to the model (Model B), these associations remained significant, with slightly larger effect sizes.

**TABLE 2:** Multinomial logistic regression model results for the association between educational failure and conduct problems trajectories

Exposure	Conduct problem trajectories									
	Childhood‐limited versus low		Adolescent‐onset versus low		Early‐onset persistent versus low		Early‐onset persistent versus childhood‐limited		Early‐onset persistent versus adolescent‐onset	
	OR_adj_ (95% CI)	*p‐*value	OR_adj_ (95% CI)	*p‐*value	OR_adj_ (95% CI)	*p‐*value	OR_adj_ (95% CI)	*p‐*value	OR_adj_ (95% CI)	*p‐*value
School failure, yes (*n* = 3018)										
Model A	1.57 (1.21–2.03)	.001	1.96 (1.39–2.75)	<.001	2.99 (1.85–4.83)	<.001	1.91 (1.17–3.09)	.009	1.53 (0.89–2.62)	.125
Model B	1.83 (1.37–2.44)	<.001	2.17 (1.52–3.09)	<.001	3.58 (2.09–6.14)	<.001	1.96 (1.18–3.27)	.010	1.65 (0.93–2.94)	.087
Education‐PRS *z*‐score (*n* = 3469)										
Model C	1.15 (1.01–1.30)	.040	1.21 (1.00–1.47)	.045	1.31 (1.01–1.70)	.044	1.14 (0.87–1.51)	.347	1.08 (0.79–1.47)	.635

*Note*: 2004 Pelotas (Brazil) Birth Cohort. Model A: Adjusted by sex of the child, family income, maternal schooling, maternal depression, school environment score, and child neurocognitive indicators (low child development, low IQ, and attention problems). Model B: Adjusted by sex of the child, family income, maternal schooling, maternal depression, school environment score, child neurocognitive indicators (low child development, low IQ, and attention problems), and behaviour problems at age 4‐years. Model C: Adjusted by family income, maternal schooling, school environment score, sex of the child, and 10 first principal components for genetic ancestry.

Abbreviations: 95% CI, 95% confidence interval; education‐PRS, education polygenic risk score; OR, odds ratio; *P*_*T*_, *P*‐value threshold for the PRS.

Table 2 also shows adjusted associations between education‐PRS (in standard deviation units) and conduct problem trajectories (Model C). Children with higher education‐PRS had increased odds of being on the childhood‐limited (OR: 1.15; 95% CI: 1.01; 1.30), adolescence‐onset (OR: 1.21; 95% CI: 1.00; 1.47), and early‐onset persistent groups (OR: 1.31; 95% CI: 1.01; 1.70), compared to the low group (all *p* < .05). In contrast to the model examining school failure, there was no evidence that education‐PRS differentiated the early‐onset persistent trajectory from the childhood‐limited or adolescence‐onset groups.

### Are there different effects of school failure and education‐PRS in different social environments?

Next, we examined whether associations between school failure and education‐PRS and conduct problem trajectories varied according to family and school environments. This was done separately for each indicator of education in relation to a broad measure of the family income and school environment.

The most consistent and strong variations in the association between both school failure and education‐PRS and conduct problems trajectories were observed for the school environment variable (see Table 3). First, using the carer‐reported measure of school failure, the associated risk for childhood‐limited, adolescence‐onset, and early‐onset persistent groups (compared to the low group) was significantly larger among children in more favourable versus less favourable school environments (1st vs. 3rd tertile). For example, among children in the less favourable school environment, repeating a grade increased the odds for early‐onset persistent problems 2‐fold (OR: 2.20; 95% CI: 1.03; 4.70), whereas, for children in the most favourable school environment, there was a 3‐fold increase (OR: 3.12; 95% CI: 1.15; 8.47; *p*‐value for interaction = .014). Similar results were observed when using the education‐PRS exposure variable. More precisely, compared to children in the worst school environment, the association of the education‐PRS with conduct problem trajectories was significantly stronger for children in the most favourable school environment. Thus, both school failure and education‐PRS measures suggest that the association between school performance and conduct problem trajectories is larger for children in better school environments.

**TABLE 3:** Moderator effects for the association between educational performance and child conduct problem trajectories

Exposure		Outcome: Conduct problem trajectories					
	Moderator	Childhood‐limited versus low		Adolescent‐onset versus low		Early‐onset persistent versus low	
		OR (95% CI)	*p*‐value interaction	OR (95% CI)	*p*‐value interaction	OR (95% CI)	*p*‐value interaction
School failure	School environment score^a^ ^,^ ^b^						
	1st (better)	1.41 (0.87; 2.28)	Ref.	2.42 (1.25; 4.68)	Ref.	3.12 (1.15; 8.47)	Ref.
	2nd	2.17 (1.33; 3.53)	.841	3.63 (1.84; 7.15)	.847	3.09 (1.12; 8.51)	.266
	3rd (worse)	1.27 (0.85; 1.90)	.170	1.14 (0.70; 1.86)	.040	2.20 (1.03; 4.70)	.014
Education‐PRS z‐score	School environment score^a^ ^,^ ^c^						
	1st (better)	1.45 (1.14; 1.83)	Ref.	1.59 (1.10; 2.29)	Ref.	1.48 (0.89; 2.47)	Ref.
	2nd	1.17 (0.93; 1.46)	.111	1.36 (0.92; 2.00)	.938	2.48 (1.39; 4.41)	.894
	3rd (worse)	0.91 (0.73; 1.14)	.015	1.02 (0.77; 1.36)	.010	1.02 (0.68; 1.53)	.006

*Note*: 2004 Pelotas (Brazil) Birth Cohort.

Abbreviations: 95% CI, 95% confidence interval; education‐PRS, education polygenic risk score; OR, odds ratio.

^a^ In tertiles.

^b^ Adjusted by sex of the child, family income, maternal schooling, maternal depression, and child neurocognitive indicators (low child development, low IQ, and attention problems).

^c^ Adjusted by family income, maternal schooling, sex of the child, and 10 first principal components for genetic ancestry.

However, interaction results for family income showed little evidence. Considering education‐PRS, its association with childhood‐limited problems was higher in mid‐income compared to low‐income families (2nd vs. 1st tertile; *p* = .024), but no other interaction for family income was found either for observed or education‐PRS measures (see Table S3).

## DISCUSSION

This population‐based, longitudinal study in Brazil showed one in four children experienced school failure (repeating a grade) up to age 11 years and school failure was strongly associated with conduct problems persisting through childhood into adolescence. Smaller associations were also observed with childhood‐limited and adolescence‐onset groups. These findings were consistent when analysed using two different approaches, considering both school failure measured via self‐report and an education‐PRS, with triangulation across these methods helping to strengthen causal inference. Interestingly, the association between school failure and conduct problems trajectories was larger for children in better school environments; no variation in effects was found according to family socioeconomic factors.

The prevalence of school failure (one quarter repeating at least one grade by age 11) in the current study was similar to that previously reported in South Brazil (Cunha et al., 2019). Studies involving conduct problems trajectories from Brazil are scarce and we are not aware of any other study in Brazil (or other low‐ and middle‐income country) that has delineated conduct problem trajectories from early childhood through adolescence. It is very difficult to know whether differences in rates of different conduct problem trajectories between studies reflect methodological differences or real variation across populations. However, we found a high proportion of children following a low conduct problems trajectory (71.2%) compared to studies in high‐income countries (3–17 years; rates ranging from 48.0% to 64.3%; Barker & Maughan, 2009; Gutman et al., 2019; Sentse et al., 2017). And there was a low prevalence of children in both the early‐onset persistent group (3.9%) and adolescent‐onset group (7.3%) in our study, compared to in several studies in HICs: early‐onset persistent rates ranging from 4.0% to 25.0% (Barker & Maughan, 2009; Bauer et al., 2021; Gutman, 2019; Gutman et al., 2019; Li, 2017; Odgers et al., 2007; Sentse et al., 2017; Shaw et al., 2019); adolescent‐onset group rates ranging from 11.8% to 15.0% (Barker & Maughan, 2009; Bauer et al., 2021; Sentse et al., 2017). However, the size of the childhood‐limited group (17.7%) in our study is within the range of several others in Europe: 12.0%–15.9% (Bauer et al., 2021; Sentse et al., 2017).

Prior longitudinal studies have found inconsistent and often weak results for the relationship between educational performance and conduct problems when adjusting for child IQ or language skills (Kulkarni et al., 2021). All these studies were conducted in North America, the UK, and New Zealand, so the associations found in our Brazilian study may reflect a stronger association in that social context. The fact that children with poor performance must repeat school grades in Brazil (24.2% by age 11 in our cohort) could be important in that regard. Children held back a year must transition to a new class of younger children, undermining bonding with peers who have managed to progress, and tying them closer to other children who are held back. Thus, a policy of repeating grades may selectively group children closer together who have more difficulties at school, as well as challenge children's confidence about their academic abilities and capacity to succeed. The significant associations in the current study might also reflect, unlike in most prior studies, our use of conduct problem trajectories as an outcome, where different trajectories have specific psychosocial determinants (Fairchild et al., 2013; Moffitt, 2018). Indeed, two cohorts in the UK and New Zealand, which did examine longitudinal trajectories as an outcome (and also used education‐PRS), also found that education indicators (including genetic) were most strongly related to early‐onset persistent group, rather than other trajectories (Wertz et al., 2018). We emphasise again that, in this and our study, the association could reflect a cumulative impact of poor school performance and conduct behaviours reinforcing each other, as well as unidirectional effects.

The current study showed that the association between educational performance and conduct problem trajectories varied by school context. Pelotas, like the rest of Brazil, is a city with considerable socioeconomic inequality and markedly different school environments. Perhaps surprisingly, repeating a grade (and having a high education‐PRS) was associated with conduct problems most strongly for children in better schools (in terms of social support and less violence). These results could be explained by negative labelling effects that override any protective influence of more favourable school environments on conduct problems, when grade repetition occurs. According to labelling theory, escalating behaviour problems arise when individuals internalise social reactions that communicate, they have deviated from social norms and expectations (Farrington & Murray, 2014). This labelling hypothesis was also suggested by another study identifying poor academic performance as a determinant of conduct problems, in which the authors considered that different expectations by teachers, students, and parents tended to follow school failure, and children then became less motivated to engage in school, and more prone to behaviour problems (Eisenberg et al., 2009). However, we cannot rule out the possibility that our findings stem from residual confounding. Specifically, it is possible that, in better school environments, children are less likely to repeat a grade (note rates of 18% vs. 35.4%, comparing children in schools classified as in the top vs. bottom terciles, *p* < .001), unless they have particularly high levels of personal difficulties (including possible behavioural difficulties). If this were true, the stronger association between repeating a grade and behaviour problems in better school environments could reflect a stronger child predisposition for conduct problems in that setting. In other words, the stronger effects of grade repetition in better school environments could represent a more active or evocative gene‐environment correlation in that setting. This alternative explanation is partly mitigated by adjustment for a preschool measure of child behaviour, but there could still be residual confounding.

The policy and practice implications of the link between educational performance and conduct problems depend to some extent on how much the association reflects a unidirectional or bidirectional relationship. However, early interventions supporting child development have shown benefits for both outcomes, as famously demonstrated in the Perry Preschool trial. This project reported lasting beneficial effects of preschool education in terms of improving achievement during the school years and decreasing delinquency and crime, for black children with low family socioeconomic status, who were considered at risk of failing at school (Schweinhart et al., 1985). These findings, and those from other studies (Heckman et al., 2010), suggest a key strategy tackle both educational and behavioural challenges is to invest in preschool enrichment programmes. However, for children who do develop difficulties at school, alternative measures to support learning, other than repeating grades could mitigate labelling effects and reduce subsequent social and behavioural problems. A recent initiative in Pelotas city where the current study was conducted, known as *Construindo Saberes* (Constructing Knowledge), aims to minimise the problem of age/grade distortion at school by offering extra Portuguese and math lessons to improve school performance and, subsequently, school dropout (Borges et al., 2020). School‐based programs addressing behavioural problems can complement support for parents to manage behavioural difficulties at home. The evidence‐based Incredible Years programme, which includes modules for both parents and teachers is particularly relevant in this regard (Webster‐Stratton et al., 2008).

As far as we know, this is the first study examining the association between educational attainment and conduct problem trajectories across different school environments using a PRS approach. Education‐PRS as a point of triangulation to compare with results from traditional analyses and more clearly meets the first criterion for causality of temporal precedence before the outcome. It is also plausible that an education‐PRS is less confounded than a measure of actual educational performance for analysing conduct problems. However, education‐PRS could also influence conduct problems through pathways other than educational performance, meaning that associations may not reflect a true causal effect of education. We addressed this in sensitivity analyses, by adjusting for children's baseline behaviour, and found similar results. The consistency of results based on the education‐PRS and regression models analysing carer‐reported school failure helps bolster confidence in the findings. The education‐PRS in this study explained a small (1.0%) amount of variance in observed school performance, which is similar to in previous education‐PRS studies (Wertz et al., 2018). Nonetheless, the education‐PRS was still associated with adolescent‐limited and early‐onset persistent conduct problems (compared to low‐conduct problems). Like in the observational analyses based on actual school failure, the education‐PRS was associated with a higher risk for early‐onset persistent problems compared to adolescent‐onset problems; however, this was not significant, perhaps because of low power for the education‐PRS which explained just 1% of the variance in educational performance.

The limitations of the study are as follows. First, although the emphasis of this study is on the possible role of school failure on behaviour, reverse causality is also plausible. Indeed, a reinforcing cascade of school difficulties and behavioural problems may be particularly important for persistent conduct problems (Moffitt, 1993b). Adjusting for a measure of pre‐school behaviour helped specify the direction of effects, as did the use of an education‐PRS, but part of the association may still reflect the impact of behavioural difficulties on school learning. Second, there was a large amount of missing data at the age of 15 years given the Covid‐19 pandemic outbreak during the fieldwork, and there were some differences between the participants included and those not included in the analysis. Although previous sensitivity analysis showed a similar number, shapes, and prevalence of conduct problems trajectories for those with complete 15‐years assessments (data not shown), still the results are not necessarily representative of the total population. Third, the measure used to assess the school environment, despite being composed of a set of items broadly representing the proposed objective, has not been fully validated and further studies are needed to further investigate its psychometric properties. Also, we didn't include information from the father, including education and mental health, that could impact the children's outcomes. Fourth, the education‐PRS has only been previously tested in European ancestry populations. The current study is a mixed population, and several genetic factors may vary across ancestral populations (Grinde et al., 2019). However, we document a positive association between the education‐PRS and carer‐reported school failure in our sample, suggesting the robustness of the score, and we adjusted for ancestry in the analyses. The fact of gene‐environment correlation (education‐PRS being associated with actual school failure) was key to our genetic study design, to consider if educational failure contributed to conduct problems. We also adjusted for pre‐school conduct problems to reduce the chance that any association with conduct problems reflects other paths between education‐PRS and the outcome. However, we cannot rule out that the education‐PRS was associated either with other genetic variants related to conduct problems or that the education‐PRS has evocative or active correlations with other environmental factors influencing conduct problems that were not adjusted for in this study.

In conclusion, this multi‐method study suggests that educational performance and conduct problems are intimately entwined, particularly when children demonstrate behavioural problems spanning from childhood into adolescence. This is the first study to suggest that school context matters, such that repeating a grade in a ‘better’ school can have worse consequences than in other school contexts where repeating a grade is more normative. Future studies using an education‐PRS, testing person‐environment interactions, and outcomes delineating longitudinal trajectories are needed to confirm these results. However, early prevention programs that show benefit for both academic and behavioural difficulties are available, and educators need to address the potential social consequences of poor educational performance when it does occur, particularly by reviewing policies of grade retention which may significantly contribute to stigma and peer influences implicated in the development and persistence of conduct problems, with multiple individual and social costs through the life‐course.

## AUTHOR CONTRIBUTIONS

**Thais Martins‐Silva**: Conceptualization; Formal analysis; Methodology; Writing – original draft; Writing – review & editing. **Andreas Bauer**: Methodology; Writing – review & editing. **Alicia Matijasevich**: Writing – review & editing. **Iná S. Santos**: Writing – review & editing. **Aluísio Barros**: Writing – review & editing. **Ulf Ekelund**: Writing – review & editing. **Luciana Tovo‐Rodrigues**: Methodology; Writing – review & editing. **Joseph Murray**: Conceptualization; Methodology; Supervision; Writing – original draft; Writing – review & editing.

## CONFLICT OF INTEREST

The authors have declared that they have no competing or potential conflicts of interest.

## ETHICAL CONSIDERATIONS

The 2004 Pelotas Birth Cohort study obtained ethical approval from the Medical School Ethics Committee of the Federal University of Pelotas. Mothers were fully informed of all follow‐up procedures, the general objectives, the voluntary nature of their participation, their right to not participate, their right to not answer specific questions, and their right to the confidentiality of the information provided.

## Supporting information

Supporting Information S1

## Data Availability

Data available on request from the authors.
