# Supplementary material for: Educational performance and conduct problem trajectories from childhood to adolescence: Observational and genetic associations in a Brazilian birth cohort
Source: JCPP Adv. 2022 Oct 17;2(4):e12105. doi: 10.1002/jcv2.12105 (PMC10242956; doi:10.1002/jcv2.12105)
Supplement: Supplementary file 1 — Supporting Information S1 [file JCV2-2-e12105-s001.docx]

**SUPPORTING INFORMATION**

**EDUCATIONAL PERFORMANCE AND CONDUCT PROBLEM TRAJECTORIES FROM CHILDHOOD TO ADOLESCENCE: OBSERVATIONAL AND GENETIC ASSOCIATIONS IN A BRAZILIAN BIRTH COHORT**

*Thais Martins-Silva ^1,2^, Andreas Bauer ^1,2^, Alicia Matijasevich ^2,3^, Iná Santos ^2,4^, Aluísio Barros ^2,5^, Ulf Ekelund ^6,7^, Luciana Tovo-Rodrigues ^1,2^, Joseph Murray ^1,2^*

^1^ Human Development and Violence Research Centre (DOVE), Federal University of Pelotas, Pelotas, Brazil

^2^ Post-graduate Program in Epidemiology, Federal University of Pelotas, Pelotas, Brazil

^3^ Departamento de Medicina Preventiva, Faculdade de Medicina FMUSP, Universidade de São Paulo, SP, Brasil

^4^ Postgraduate Program in Pediatrics and Child Health, School of Medicine, Pontifical Catholic University of Rio Grande do Sul, Porto Alegre, Brazil

^5^ International Center for Equity in Health, Federal University of Pelotas, Pelotas, Brazil

^6^ Department of Sport Medicine, Norwegian School of Sport Sciences, Oslo, Norway

^7^ Department of Chronic Diseases and Ageing, Norwegian Institute of Public Health, Oslo, Norway

**SUPPLEMENTARY METHODS**

*2004 Pelotas (Brazil) Birth Cohort*

The 2004 Pelotas (Brazil) Birth Cohort is a population-based, prospective longitudinal study, including all children born in 2004 in Pelotas, a southern Brazilian city of approximately 340,000 people. All women with live births residing in the urban area of the city (*n*= 4,261) were invited to participate. Of those women, 4,231 (99.3%) gave informed consent and were included in the study with their children. Trained interviewers collected information on maternal and child health outcomes within 24 hours after delivery using a structured questionnaire, and all newborns were examined by a pediatrician. Since the perinatal visit, mothers and their children have been assessed again at 3, 12, 24, and 48 months at home, and at 6 and 11 years at a research clinic run by the Postgraduate Program in Epidemiology at the Federal University of Pelotas. At the age of 15 years, follow-up assessments were completed in the research clinic as well as via telephone due to the Covid-19 pandemic, which interrupted in-person assessments in March 2020 after about half of the cohort had been interviewed (n=2,029; 50.4%). Mental health outcomes were measured by psychologists and RedCap was used as the instrument for data collection (Harris et al., 2009).

DNA samples

The saliva samples for the DNA analyses were collected at the age of 6 years using the DNA Oragene Genotek® – 250 collection kit (Santos et al., 2014). Genomic DNA was extracted following the manufacturer’s instructions. DNA was quantified and qualified by spectrophotometry using NanoDrop. DNA samples were genotyped for approximately 600,000 SNPs using an Infinium Global Screening Array 2. Imputation of remaining non-genotyped variants was performed based on data from the overall population of the 1000 Genomes Project (phase 3) as a reference panel (Abecasis et al., 2012), using SHAPEIT 2 (O’Connell et al., 2014) and MINIMAC3 (Delaneau et al., 2013).

Before creating the education-PRS, genotyped and imputed autosomal variants were filtered to include only those with: < 2% missing genotypes, minor allele frequency ≥ 0.01, imputation quality (r^2^) > 0.3, and a Hardy-Weinberg equilibrium deviation with *p*-value > 1×10^-6^. After the application of the quality control filters, 11,811,746 variants were retained. The major histocompatibility complex (MHC) region (chr6: 26–33M) was removed and independent SNPs per 250-kb window and r^2^ = 0.1 were included. The PRS was constructed using PRSice 2.2.

*Peace Zone questionnaire*

The Peace Zone questionnaire (Prothrow-Stith, Chéry, & Oliver, 2001) was used to assess the school environment, including the following 11 items concerning the child’s school, and relationships with others at school. *“Kids in my classroom push and shove each other a lot”; “Kids in my classroom yell at each other a lot”; “Kids in my class look out for each other”; “Kids in my classroom wait their turn to talk”; “I always wait my turn to talk”; “There are a lot of fights at my school”; “When I’m angry or sad, I talk about my feelings to other kids at school“; “When I’m angry or sad, I talk about my feelings to adults at school”; “I feel safe at my school”; “I feel close to people at this school”; and “I learn a lot at my school”*. The items are scored on a 4-point scale (*all of the time; most of the time; sometimes; and never*). Items on school violence (i.e., “*Kids in my classroom push and shove each other a lot”*; *“Kids in my classroom yell at each other a lot”; and “There are a lot of fights at my school”*) were reverse-coded and then all items were summed to create a total school environment score – ranging from 0 to 33 and indicating a more supportive, less violent school environment.

**References**

Abecasis, G. R., Auton, A., Brooks, L. D., DePristo, M. A., Durbin, R. M., Handsaker, R. E., ... & McVean, G. A. (2012). An integrated map of genetic variation from 1,092 human genomes. *Nature*, *491*(7422), 56–65. <https://doi.org/10.1038/nature11632>

Delaneau, O., Zagury, J.-F., & Marchini, J. (2013). Improved whole-chromosome phasing for disease and population genetic studies. In *Nature methods* (Vol. 10, Issue 1, pp. 5–6). <https://doi.org/10.1038/nmeth.2307>

Harris PA, Taylor R, Thielke R, Payne J, Gonzalez N, Conde JG. Research electronic data capture (REDCap)—A metadata-driven methodology and workflow process for providing translational research informatics support. J Biomed Inform [Internet]. 2009;42(2):377–81. Available from: https://www.sciencedirect.com/science/article/pii/S1532046408001226

O’Connell, J., Gurdasani, D., Delaneau, O., Pirastu, N., Ulivi, S., Cocca, M., … & Marchini, J. (2014). A general approach for haplotype phasing across the full spectrum of relatedness. *PLoS Genetics*, *10*(4), e1004234. <https://doi.org/10.1371/journal.pgen.1004234>

Prothrow-Stith, D., Chéry, J. M., & Oliver, J. (2001). PeaceZone: A program for teaching social literacy. *Oxford: Harvard University School of Public Health, Division of Public Health Practice.*

Santos, I. S., Barros, A. J. D., Matijasevich, A., Zanini, R., Cesar, M. A. C., Camargo-Figuera, F. A., ... & Victora, C. G. (2014). Cohort profile update: 2004 Pelotas (Brazil) Birth Cohort Study. Body composition, mental health and genetic assessment at the 6 years follow-up. *International Journal of Epidemiology*, *43*(5), 1437-1437f. <https://doi.org/10.1093/ije/dyu144>

**Table S1.** Model fit statistics for conduct problem trajectories. 2004 Pelotas (Brazil) Birth Cohort.

| **Conduct problem trajectory** | **n (%)** | **APP (sd)** | **Parameter** | **β** | **SE** | ***p*-value** |
| --- | --- | --- | --- | --- | --- | --- |
| Early-onset persistent | 150 (3.8) | 0.877 (0.168) | Intercept | -2.923 | 0.812 | *<0.001* |
|  |  |  | Linear | 1.836 | 0.311 | *<0.001* |
|  |  |  | Quadratic | -0.188 | 0.036 | *<0.001* |
|  |  |  | Cubic | 0.006 | 0.001 | *<0.001* |
| Adolescence-onset | 286 (7.3) | 0.828 (0.181) | Intercept | 7.720 | 0.631 | *<0.001* |
|  |  |  | Linear | -3.068 | 0.243 | *<0.001* |
|  |  |  | Quadratic | 0.379 | 0.028 | *<0.001* |
|  |  |  | Cubic | -0.013 | 0.001 | *<0.001* |
| Childhood-limited | 697 (17.7) | 0.776 (0.169) | Intercept | -2.392 | 0.553 | *<0.001* |
|  |  |  | Linear | 1.356 | 0.216 | *<0.001* |
|  |  |  | Quadratic | -0.167 | 0.024 | *<0.001* |
|  |  |  | Cubic | 0.006 | 0.001 | *<0.001* |
| Low | 2.805 (71.2) | 0.930 (0.121) | Intercept | 0.012 | 0.166 | 0.941 |
|  |  |  | Linear | -0.152 | 0.063 | *0.015* |
|  |  |  | Quadratic | 0.016 | 0.007 | *0.021* |
|  |  |  | Cubic | -0.001 | 0.0002 | *0.033* |

SD: Standard deviation; SE: Standard error; β: Beta coefficient; APP: average posterior probability.

| **Table S2.** Binary logistic regression model results for the association between the education-PRS and related school failure at 11-years. 2004 Pelotas (Brazil) Birth Cohort. | | | | | |
| --- | --- | --- | --- | --- | --- |
|  | **Related school failure (n=3,230)** | | | | |
|  | **N SNPs** | **Pseudo-R^2^** | **OR** | **95% CI** | ***p*-value** |
| **Education-PRS z-score**^*^ | |  |  |  |  |
| P_T_ 5e-8 | 757 | 0.0067 | 1.24 | 1.14;1.36 | <0.001 |
| P_T_ 5e-6 | 1992 | 0.0079 | 1.28 | 1.17;1.40 | <0.001 |
| P_T_ 0.05 | 67668 | 0.0102 | 1.43 | 1.27;1.60 | <0.001 |
| P_T_ 0.5 | 249700 | 0.0082 | 1.36 | 1.22;1.53 | <0.001 |
| SNP: single nucleotide polymorphisms; Education-PRS: Education polygenic risk score; PT: P-value threshold for the PRS; OR: odds ratio; 95% CI: 95% confidence interval; ^*^Adjusted by sex and 10 first principal components for genetic ancestry; After False discovery rate (FDR) correction, the p-value for an association between education-PRS and educational failure remained significant (p=0.001). | | | | | |

| **Table S3.** Adjusted linear regression model results for the association between education-PRS as exposure and conduct problems at 4-years. 2004 Pelotas (Brazil) Birth Cohort. | | | | |
| --- | --- | --- | --- | --- |
|  | **Conduct problems at 4-years** | | |  |
|  | **N** | **β_adj_ (95% CI) ^*^** | ***p*-value** | ***p*-value after FDR correction** |
| **Education-PRS z-score*** | |  |  |  |
| P_T_ 5e-8 | 3,346 | -0.003 (-0.008; 0.002) | 0.224 | 0.280 |
| P_T_ 5e-6 | 3,346 | 0.001 (-0.004; 0.006) | 0.666 | 0.740 |
| P_T_ 0.05 | 3,346 | 0.006 (0.002; 0.009) | 0.001 | 0.002 |
| P_T_ 0.5 | 3,346 | 0.005 (0.002; 0.009) | 0.005 | 0.008 |
| Education-PRS: Education polygenic risk score; PT: P-value threshold for the PRS; β: beta coefficient; 95% CI: 95% confidence interval; FDR: False discovery rate; * Adjusted by sex and 10 first principal components for genetic ancestry | | | | |

| **Table S4.** Moderator effects for the association between educational performance and child conduct problem trajectories. 2004 Pelotas (Brazil) Birth Cohort. | | | | | | | |
| --- | --- | --- | --- | --- | --- | --- | --- |
|  |  | **Outcome: Conduct Problem Trajectories** | | | | | |
| **Exposure** | **Moderator** | **Childhood-limited *vs.* Low** | | **Adolescent-onset *vs.* Low** | | **Early-onset persistent *vs.* Low** | |
|  |  | **OR (95% CI)** | ***p*-value interaction** | **OR (95% CI)** | ***p*-value interaction** | **OR (95% CI)** | ***p*-value interaction** |
| ***Related school failure*** | **Family income** ^¥.€^ |  |  |  |  |  |  |
|  | 1^st^ (poorest) | 1.48 (1.02; 2.16) | *Ref.* | 1.75 (1.10; 2.79) | *Ref.* | 2.60 (1.32; 5.14) | *Ref.* |
|  | 2^nd^ | 1.78 (1.16; 2.74) | 0.491 | 2.56 (1.46; 4.50) | 0.176 | 5.16 (2.02; 13.17) | 0.109 |
|  | 3^rd^ (richest) | 1.38 (0.74; 2.58) | 0.868 | 1.60 (0.63; 4.10) | 0.913 | 1.16 (0.30; 4.43) | 0.620 |
| ***Education-PRS***  ***z-score*** | **Family income** ^¥, £^ | |  |  |  |  |  |
|  | 1^st^ (poorest) | 0.75 (0.61; 0.91) | *Ref.* | 0.77 (0.58; 1.01) | *Ref.* | 0.64 (0.46; 0.90) | *Ref.* |
|  | 2^nd^ | 1.11 (0.89; 1.37) | **0.024** | 0.97 (0.69; 1.37) | 0.451 | 0.84 (0.51; 1.38) | 0.283 |
|  | 3^rd^ (richest) | 0.91 (0.71; 1.16) | 0.620 | 0.67 (0.44; 1.01) | 0.405 | 0.56 (0.29; 1.09) | 0.747 |
| Education-PRS: Education polygenic risk score; OR: odds ratio; 95% CI: 95% confidence interval; ^¥^ in tertiles; ^€^ Adjusted by sex of the child, family income, maternal schooling, maternal depression, and child neurocognitive indicators (low child development, low IQ, and attention problems); ^£^ Adjusted by family income, maternal schooling, sex of the child, and 10 first principal components for genetic ancestry. | | | | | | | |
